# Association between gut microbiota and gastric cancers: a two-sample Mendelian randomization study

**DOI:** 10.3389/fmicb.2024.1383530

**Published:** 2024-04-02

**Authors:** Yuan Chang, Guanzhuang Gao, Cuncheng Feng

**Affiliations:** ^1^Department of Anorectal Surgery, The Affiliated Changzhou No. 2 People’s Hospital of Nanjing Medical University, Changzhou, China; ^2^Department of Gastrointestinal Surgery, The Affiliated Changzhou No. 2 People’s Hospital of Nanjing Medical University, Changzhou, China

**Keywords:** association, gut microbiota, gastric cancer, GWAS, Mendelian randomization

## Abstract

**Background:**

Gastric cancer (GC) is the fifth most commonly diagnosed cancer worldwide, with its etiology attributed to a complex interplay of genetic, dietary, environmental factors, and infections such as *Helicobacter pylori*. Despite the known risk factors, the role of gut microbiota in the development of gastric cancer remains insufficiently explored. This study aims to elucidate the causal relationship between gut microbiota and gastric cancer using a two-sample Mendelian Randomization (MR) approach.

**Methods:**

Utilizing genome-wide association study (GWAS) summary data from the MiBioGen consortium and gastric cancer datasets, we selected instrumental variables for MR analysis based on their association with specific microbiota. We employed several MR methods, including inverse variance weighted (IVW), MR-Egger, weighted median, and others, to estimate the causal effects of gut microbiota diversity on the risk of developing gastric cancer.

**Results:**

Our analysis identified significant associations between certain gut microbiota and gastric cancer risk. Specifically, taxa such as *Clostridium sensustricto1* (OR = 0.540, 95%CI: 0.354–0.823, *p* = 0.004), *Actinomycetales* (OR = 0.756, 95%CI: 0.613–0.932, *p* = 0.009), *Selenomonadales* (OR = 0.816, 95%CI: 0.666–1.000, *p* < 0.05), *Negativicutes* (OR = 0.816, 95%CI: 0.666–1.000, *p* < 0.05), *Rikenellaceae* (OR = 0.863, 95%CI: 0.746–0.999, *p* = 0.048) were found to have a protective effect against gastric cancer. Conversely, an increased risk of gastric cancer was associated with the abundance of *Roseburia* (OR = 1.342, 95%CI: 1.071–1.681, *p* = 0.011), *Family XI* (OR = 1.132, 95%CI: 1.012–1.267, *p* = 0.030), and *Eubacterium brachy group* (OR = 1.207, 95%CI: 1.074–1.355, *p* = 0.002). The findings were robust across various MR methods and were not driven by any single SNP, indicating a genuine causal relationship.

**Conclusion:**

Our studies have shown that there is a causal relationship between intestinal flora and gastric cancer at the genetic level. *Clostridium sensustricto1*, *Actinomycetales*, *Rikenellaceae*, *Selenomonadales*, *Negativicutes*, and *Actinomycetaceae* as having a protective role against GC, while *Roseburia*, *Family XI*, and *Eubacterium brachy group* were associated with an increased risk.

## 1 Introduction

Gastric cancer (GC), classified fifth in incidence among global cancer diagnoses and occupying the third position as a causative factor of oncology-associated mortalities, were documented with approximately 1.08 million novel cases and accounted for 769,000 fatalities worldwide in the year 2020 (Hamashima, 2020; Sung et al., 2021). Despite extensive research, the etiology of gastric cancer remains partially understood, implicating genetic, dietary, environmental factors, *Helicobacter pylori* (Hp) infection, and precancerous lesions (chronic gastritis, gastric ulcer, gastric polyps, etc.) within a complex interaction network (Machlowska et al., 2020). Particularly, *Helicobacter pylori* (Hp) infection is identified as a principal risk factor (Collatuzzo et al., 2021; Mendes-Rocha et al., 2023). However, eradication of *H. pylori* does not fully preclude gastric carcinoma development, with only about 1% of infected patients developing the disease (Mirzaei et al., 2021). Contrary to earlier beliefs that the acidic environment of the human stomach precludes the colonization by microorganisms other than *Helicobacter pylori* (Devi et al., 2021; Zhou et al., 2024). Recent advances in sequencing technology have unveiled a diverse stomach microbiota, correlating gastric cancer with increased microbial diversity and abundance (Zhou et al., 2024).

The human microbiota, encompassing viruses, fungi, and bacteria, can undergo dysbiosis due to diets, antibiotics, microbial infections, and host genetics (Meng et al., 2018; Chattopadhyay et al., 2023). A balanced microbiota plays a protective role against cancer development, whereas dysbiosis may promote oncogenesis (Garrett, 2015; Meng et al., 2018). With advancements in the complexity and resolution of the human microbiota in recent years, the scientific community has bestowed increased attention on its role in the genesis of tumors (Cullin et al., 2021). The gastrointestinal tract serves as a crucial metabolic organ, hosting a substantial aggregation of microorganisms. The gastrointestinal tract, hosting a vast microbial community, is recognized for its metabolic significance and its interdependent relationship with human health throughout life. The gut microbiome, spanning the digestive system, is increasingly seen as a crucial ecological factor influencing human health (Adak and Khan, 2019; Chen C. et al., 2021).

Extensive research has highlighted the gut microbiota’s direct and indirect roles in gastric cancer’s onset, treatment, and prognosis. A study in China comparing the gut microbiota of 116 gastric cancer patients with 88 healthy controls found significant microbial alterations, including increased flora abundance, reduced butyrate-producing bacteria, and significant enrichments of *Lactobacillus*, *Escherichia*, and *Klebsiella* in cancer patients (Qi et al., 2019). Sarhadi et al. (2021), identified *Enterobacteriaceae* as prevalent in all gastric cancer types, suggesting its potential as a diagnostic biomarker. In addition, relevant studies have shown that certain gut bacteria produce metabolites like acetic acid and butyrate, influencing gastric carcinogenesis, while evidence suggests intestinal probiotics may mitigate inflammation, enhance immunity, promote tumor apoptosis, restore flora balance, and block cancer pathways, potentially curtailing gastric cancer progression (Hu et al., 2018; Chen Y. et al., 2021; Hou et al., 2022).

The gut microbiota’s role in host health is gaining acknowledgment, underscoring the need to connect gut flora with disease processes and to harness these insights for breakthroughs in disease prevention, diagnosis, and treatment. Currently, although there are studies related to the properties of the gut microbiota in gastric cancer patients, most of them are observational. Traditional observational studies have encountered difficulties in establishing causal relationships between gut microbiota and cancer risk because they are susceptible to confounding variables such as dietary patterns, environmental factors, and reverse causality effects (Birney, 2021; Yang et al., 2023). Hence, a robust methodology is essential for causal analysis. Mendelian Randomization (MR) analysis employs genetic variants, such as Single Nucleotide Polymorphisms (SNPs), as instrumental variables, drawing upon the principle of Mendel’s law of independent assortment (Sekula et al., 2016). This approach, which considers the genetic allocation at conception as akin to the randomized conditions found in controlled experiments, allows observational studies to address challenges like residual confounding and reverse causality, thus enhancing their reliability (Birney, 2021). Investigating the causal link between gut microbiota and gastric cancer through MR analysis is pivotal for elucidating pathogenesis and refining treatment modalities. Our study aims to elucidate this causal relationship using MR, advancing the understanding of gut microbiota’s role in gastric cancer risk.

## 2 Materials and methods

### 2.1 Study design

**Figure 1:**
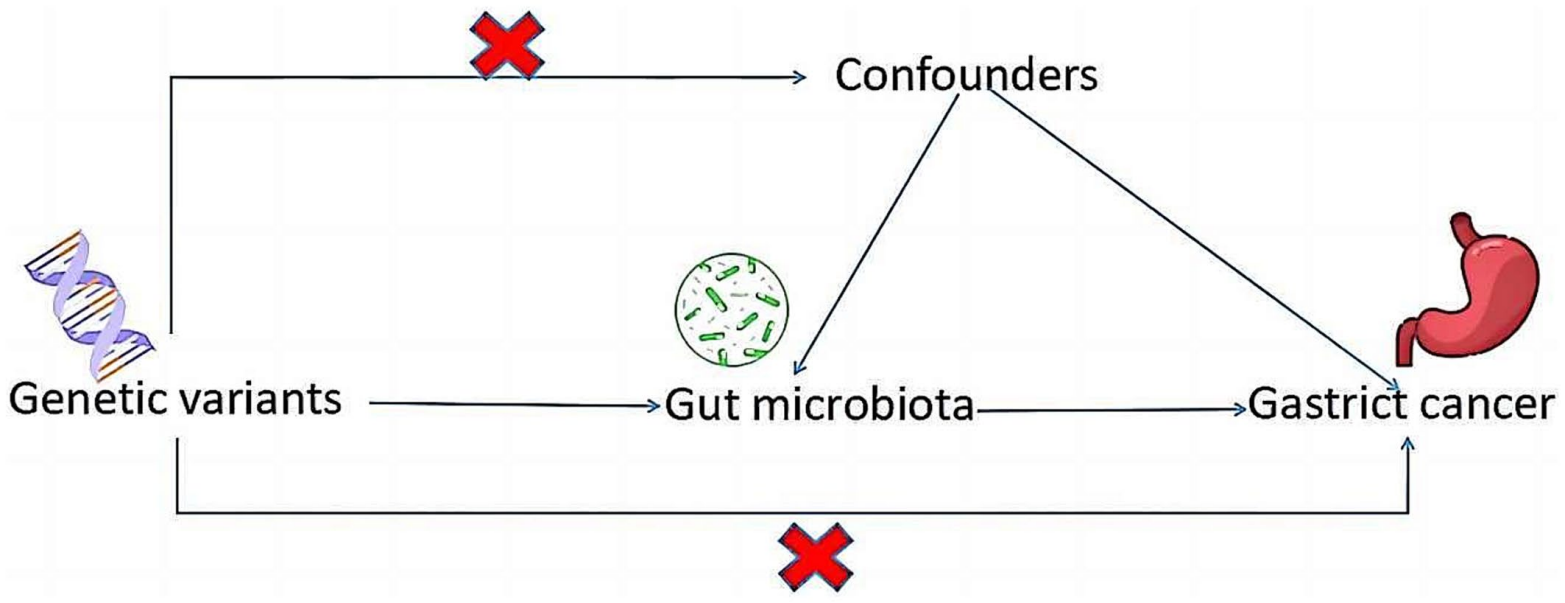
A schematic diagram illustrates the MR causality study design, elucidating the fundamental principles of MR study and the hypothetical relationship between genetic variant, exposure, and outcome.

Drawing on the genome-wide association study (GWAS) summary data for gut microbiota and GC, this investigation meticulously selected eligible instrumental variables (IVs) for Mendelian Randomization (MR) analysis to delineate the causal dynamics between gut microbiota and GC. The methodology rigorously adhered to the tripartite foundational assumptions of MR analysis: (1) The IVs identified bore a direct association with the exposure variable; (2) The IVs were not associated with any confounding factors, ensuring their independence; (3) The IVs exerted influence on the outcome exclusively through their interaction with the exposure variable (Figure 1). This study is reported following the Strengthening the Reporting of Observational Studies in Epidemiology using Mendelian Randomization guidelines is a specialized checklist for MR studies. The datasets deployed in this research are accessible publicly, so this study did not require Ethical approval or informed consent because it was derived from GWAS summary statistics.

### 2.2 Data source

GWAS summary data for gut microbiota were sourced from the MiBioGen consortium website,^1^ encompassing 14,306 samples of European descent, with informed consent obtained. The dataset included 5,594,934 SNPs for *Clostridium sensustricto1*, 5,712,148 SNPs for *Roseburia*, 5,424,038 SNPs for *Actinomycetales*, 4,330,602 SNPs for *Family XI*, 5,665,27 SNPs for *Rikenellaceae*, 5,721,008 SNPs for *Selenomonadales*, 5,221,253 SNPs for *Eubacterium brachy group*, 5,721,008 SNPs for *Negativicutes*, and 5,424,030 SNPs for *Actinomycetaceae*. In the context of gastric cancer, we analyzed summary-level data from 476,116 European individuals, which included 24,188,662 SNPs (Table 1).

**Table 1:** The GWAS datasets for exposure and outcomes.

Exposure/outcome	GWAS_ID	Consortium	Sample size	Number of SNPs	Population
Gastric cancer	ebi-a-GCST90018849	NA	476,116	24,188,662	European
Genus Clostridium sensustricto1	ebi-a-GCST90016980	NA	14,306	5,594,934	European
Genus Roseburia	ebi-a-GCST90017048	NA	14,306	5,712,148	European
Order Actinomycetales	ebi-a-GCST90017090	NA	14,306	5,424,038	European
Family Family XI	ebi-a-GCST90016938	NA	14,306	4,330,602	European
Family Rikenellaceae	ebi-a-GCST90016950	NA	14,306	5,665,279	European
Order Selenomonadales	ebi-a-GCST90017107	NA	14,306	5,721,008	European
Genus *Eubacterium brachy* group	ebi-a-GCST90016996	NA	14,306	5,221,253	European
Class Negativicutes	ebi-a-GCST90016922	NA	14,306	5,721,008	European
Family Actinomycetaceae	ebi-a-GCST90016925	NA	14,306	5,424,030	European

### 2.3 IV selection

**Figure 2:**
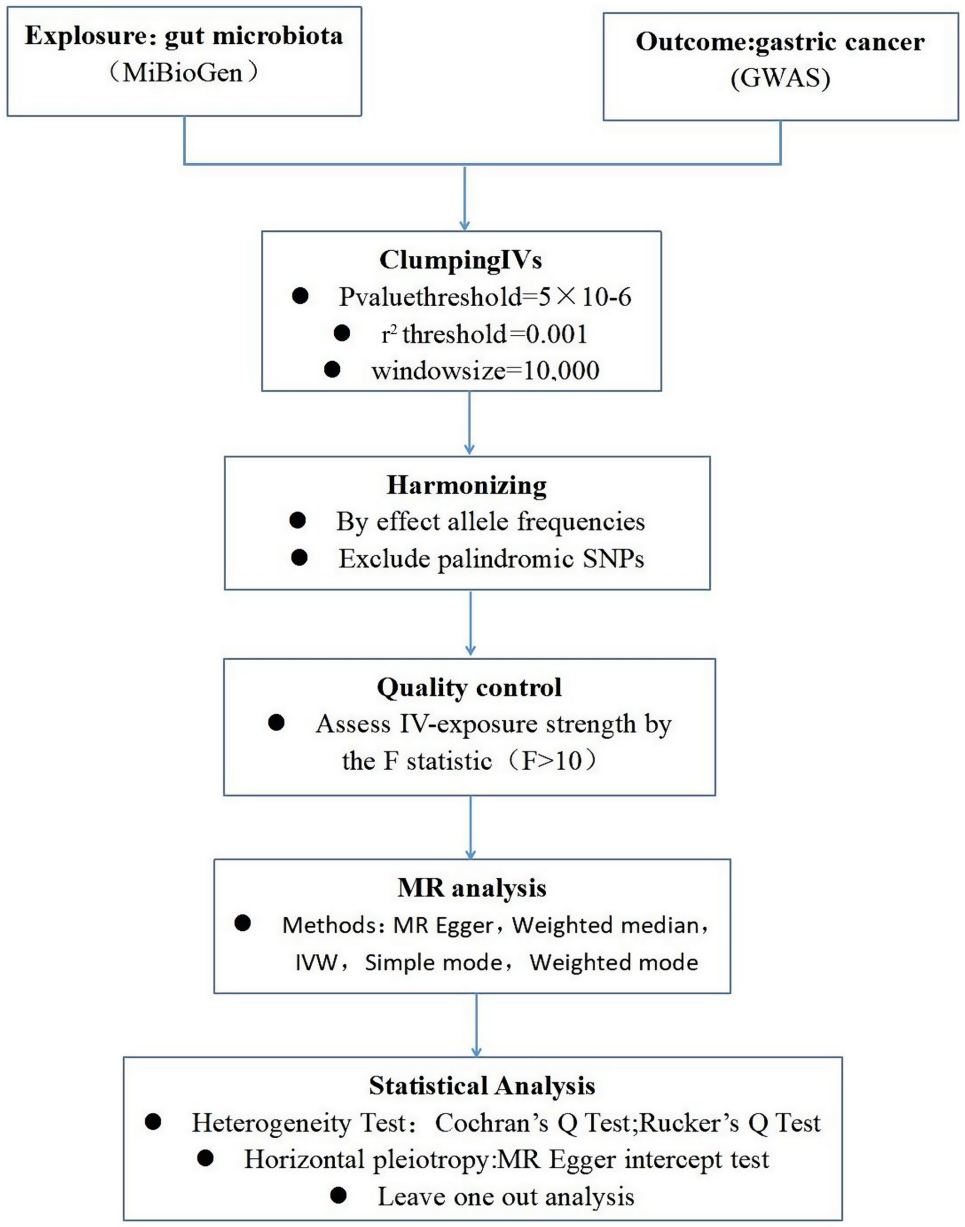
A schematic model of the Mendelian randomization (MR) study. GWAS, Genome Wide Association Studies; IV, Instrumental variable; SNP, single nucleotide polymorphism; MR, Mendelian randomization; IVW, Inverse-variance weighted.

To ensure the robustness and reliability of our MR analysis, we implemented stringent quality controls for IVs selection, adhering to the three foundational assumptions of MR analysis (Figure 1). Initially, we identified SNPs associated with nine gut microbiotas (including *Clostridium sensustricto1*, *Roseburia*, *Actinomycetales*, *Family XI*, *Rikenellaceae*, *Selenomonadales*, *Eubacterium brachy group*, *Negativicutes*, *Actinomycetaceae*) with a significance threshold of *p* < 1E-5. To mitigate the influence of linkage disequilibrium (LD), SNPs within strong LD were excluded (r^2^ < 0.001, clumping distance = 10,000 kb). Furthermore, only SNPs with an F-statistic >10 were selected to satisfy the criterion for a strong association with the exposure. Additionally, palindromic SNPs with intermediate allele frequencies were removed to enhance result accuracy. The F-statistic was calculated using the formula: *F = β^2^exposure/SE^2^exposure* (Burgess et al., 2017), to assess the robustness of the instrumental SNPs, considering an F-statistic >10 indicative of a minimal weak instrument bias (Papadimitriou et al., 2020; Figure 2).

### 2.4 Statistical analysis

Our MR analysis was conducted using five distinct approaches: the random-effects inverse variance weighted (IVW) method as the primary analysis, complemented by MR Egger, weighted median, simple mode, and weighted mode analyses. The random-effects IVW results served as the cornerstone of our study. To evaluate heterogeneity, we utilized the Cochran’s Q statistic for MR-IVW and Rucker’s Q statistic for MR Egger, with *p* > 0.05 indicating no significant heterogeneity (Sekula et al., 2016). The MR Egger intercept test was employed to assess horizontal pleiotropy, with *p* > 0.05 suggesting an absence of horizontal pleiotropy. Moreover, the MR-PRESSO test not only identified horizontal pleiotropy but also detected outliers. The “Leave one out” analysis was instrumental in determining if a single SNP disproportionately influenced the causal relationship between gut microbiota and GC. The global test in MR-PRESSO analysis was applied for horizontal pleiotropy assessment, and the distortion test within the same framework was utilized to ascertain the presence of outliers in our MR analysis. All Mendelian Randomization analyses were performed utilizing the ‘Two Sample MR’ (version 0.5.6) and ‘MR-PRESSO’ (version 1.0) packages in R version 4.2.3, setting statistical significance at *p* < 0.05.

## 3 Results

### 3.1 IVs selection

Through meticulous SNP screening for exposure association and linkage disequilibrium (LD) removal, we identified instrumental variables: 7 SNPs for *Clostridium sensustricto1*, 13 for *Roseburia*, 5 for *Actinomycetales*, 8 for *Family XI*, 19 for *Rikenellaceae*, 12 for *Selenomonadales*, 10 for *Eubacterium brachy group*, 12 for *Negativicutes*, and 5 for *Actinomycetaceae* (Table 2).

**Table 2:** The significant causal effect of gut microbiota on gastric cancer.

Exposure	Method	SNPs (*n*)	*p*-value	OR (95%CI)
Genus Clostridium sensustricto1	MR Egger	7	0.042	0.243 (0.087–0.676)
	Weighted median	7	0.535	0.890 (0.615–1.287)
	Inverse variance weighted	7	0.004	0.540 (0.354–0.823)
	Simple mode	7	0.951	0.983 (0.572–1.687)
	Weighted mode	7	0.955	0.986 (0.627–1.551)
Genus Roseburia	MR Egger	13	0.958	1.024 (0.424–2.473)
	Weighted median	13	0.447	1.127 (0.828–1.536)
	Inverse variance weighted	13	0.011	1.342 (1.071–1.681)
	Simple mode	13	0.464	1.198 (0.750–1.914)
	Weighted mode	13	0.643	1.107 (0.729–1.682)
Order Actinomycetales	MR Egger	5	0.884	0.926 (0.424–2.473)
	Weighted median	5	0.033	0.747 (0.828–1.536)
	Inverse variance weighted	5	0.009	0.756 (0.613–0.932)
	Simple mode	5	0.166	0.689 (0.750–1.914)
	Weighted mode	5	0.173	0.685 (0.729–1.682)
Family Family XI	MR Egger	8	0.201	1.740 (0.817–3.705)
	Weighted median	8	0.550	1.043 (0.909–1.197)
	Inverse variance weighted	8	0.030	1.132 (1.012–1.267)
	Simple mode	8	0.784	1.034 (0.820–1.305)
	Weighted mode	8	0.679	1.046 (0.853–1.283)
Family Rikenellaceae	MR Egger	19	0.477	1.233 (0.701–2.171)
	Weighted median	19	0.193	0.869 (0.704–1.073)
	Inverse variance weighted	19	0.048	0.863 (0.746–0.999)
	Simple mode	19	0.521	0.903 (0.666–1.225)
	Weighted mode	19	0.320	0.883 (0.695–1.121)
Order Selenomonadales	MR Egger	12	0.924	0.952 (0.351–2.582)
	Weighted median	12	0.443	0.899 (0.684–1.181)
	Inverse variance weighted	12	<0.050	0.816 (0.666–1.000)
	Simple mode	12	0.635	0.914 (0.638–1.311)
	Weighted mode	12	0.581	0.910 (0.656–1.261)
Genus *Eubacterium brachy* group	MR Egger	10	0.056	2.024 (1.091–3.754)
	Weighted median	10	0.341	1.087 (0.916–1.290)
	Inverse variance weighted	10	0.002	1.207 (1.074–1.355)
	Simple mode	10	0.802	1.036 (0.792–1.354)
	Weighted mode	10	0.640	1.060 (0.836–1.345)
Class Negativicutes	MR Egger	12	0.924	0.952 (0.351–2.582)
	Weighted median	12	0.446	0.899 (0.684–1.182)
	Inverse variance weighted	12	<0.050	0.816 (0.666–1.000)
	Simple mode	12	0.636	0.914 (0.637–1.312)
	Weighted mode	12	0.568	0.910 (0.663–1.248)
Family Actinomycetaceae	MR Egger	5	0.887	0.928 (0.361–2.386)
	Weighted median	5	0.050	0.747 (0.558–1.000)
	Inverse variance weighted	5	0.009	0.756 (0.613–0.932)
	Simple mode	5	0.172	0.688 (0.442–1.070)
	Weighted mode	5	0.188	0.682 (0.425–1.095)

### 3.2 MR analysis

**Figure 3:**
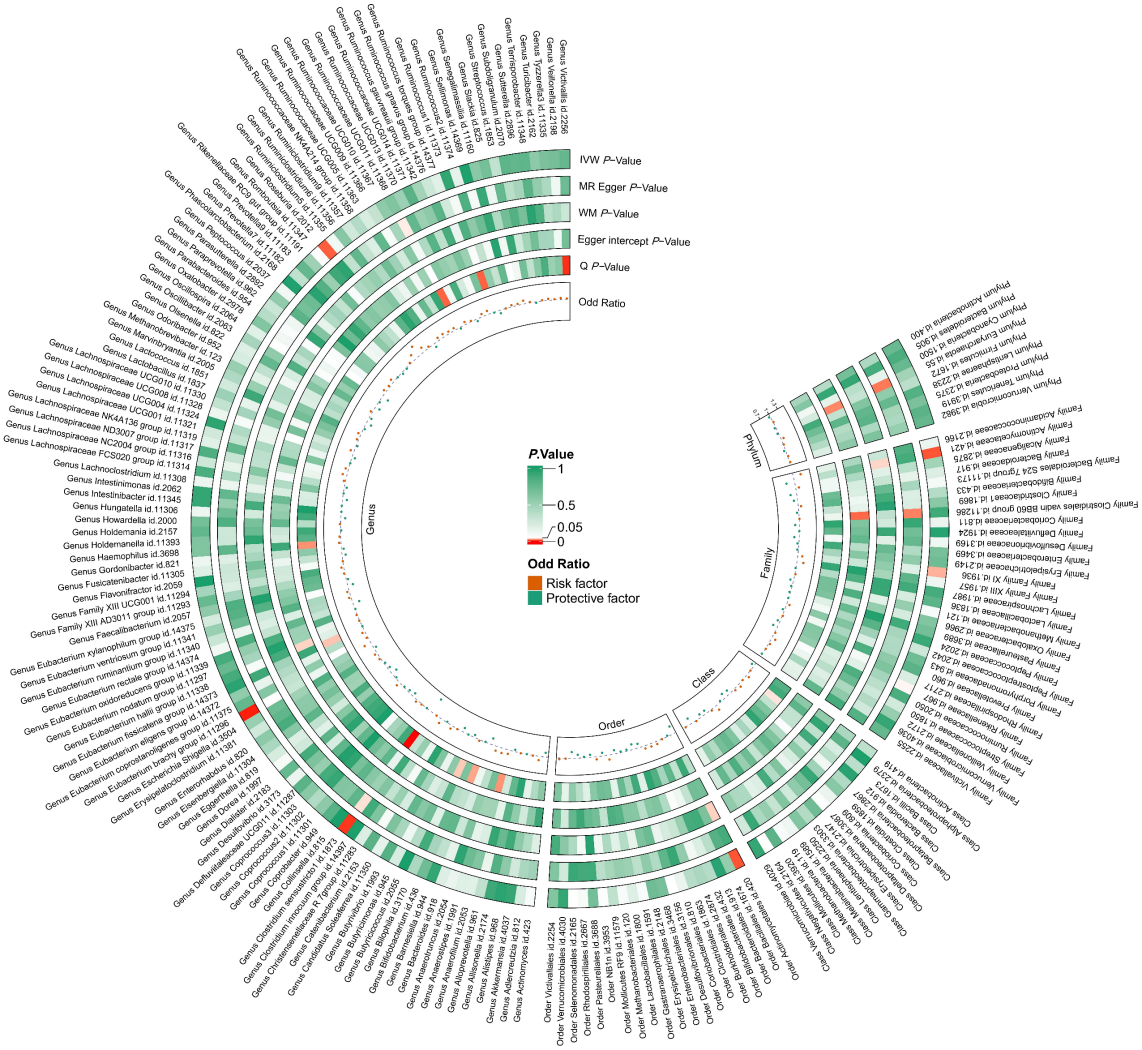
196 species of gut microbiota for their causal association with gastric cancer. There are three types of gut microbiota data missing, and a total of 193 gut flora results are shown.

**Figure 4:**
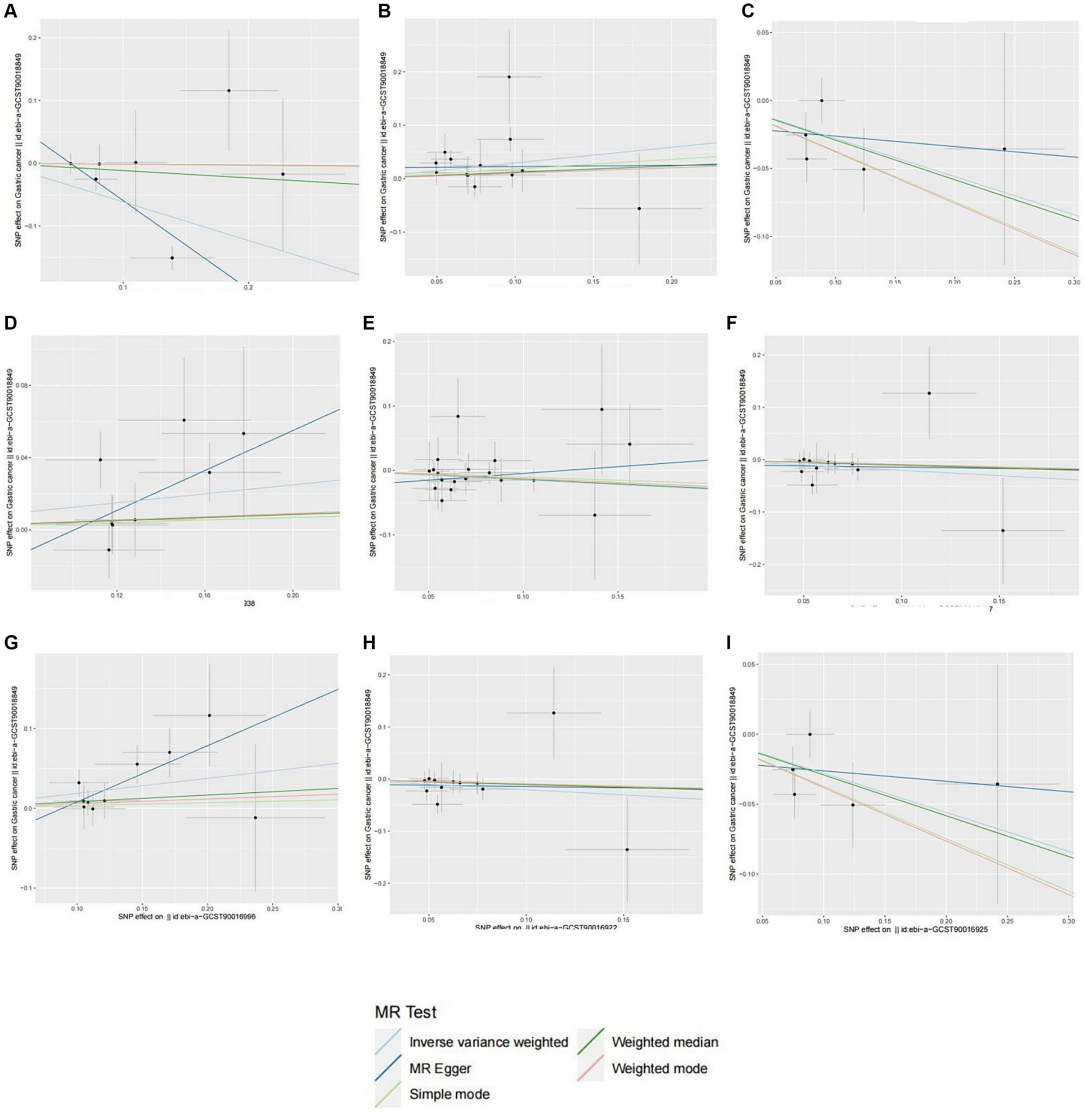
Scatter plots of different MR outcomes. Scatter plots showed the causal effect of diffrent gut microbotia on Gastric cancer. **(A)** *Clostridium sensustricto1*; **(B)** *Roseburia*; **(C)** *Actinomycetales*; **(D)** *Family X*; **(E)** *Rikenellaceae*; **(F)** *Selenomonadales*; **(G)** *Eubacterium brachy group*; **(H)** *Negativicutes*; **(I)** *Actinomycetaceae*. The slopes of each line represent the causal association for each method.

**Figure 5:**
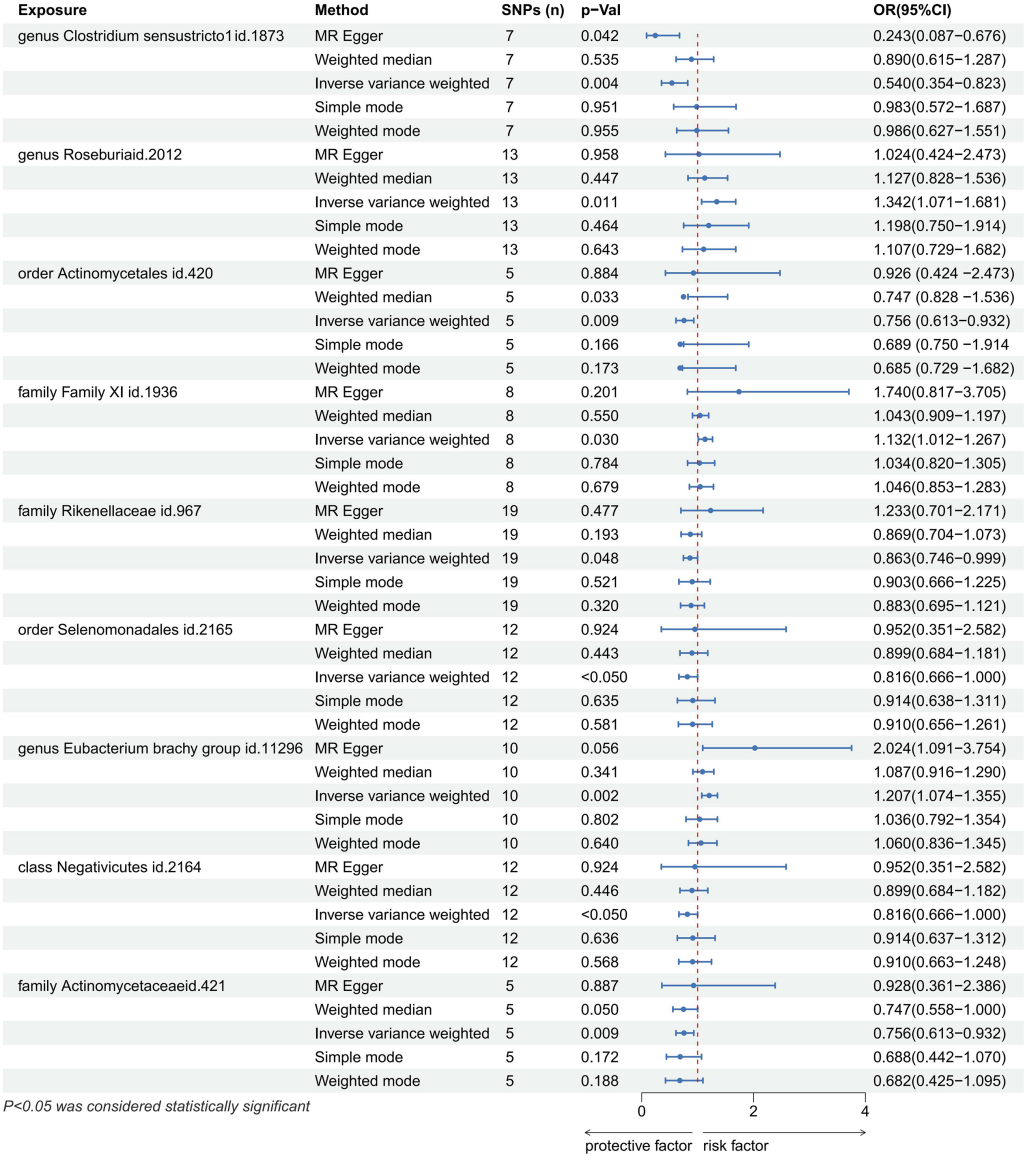
Forest plot showing the causal relationship between the genetically identified 9 microbial taxa and Gastric cancer using the MR analysis. The blue line segments and blue dots indicate the 95% CIs and OR-value for the different gut microbiota for the 5 methods (IVW, MR Egger, Weighted median, Simple mode, Weighted mode).

In total, we analyzed 196 species of gut flora for their causal association with gastric cancer (Figure 3). The inverse variance weighted (IVW) method, as our primary analysis tool, revealed a causal association between the relative abundance of nine genetically predicted bacterial taxa and gastric cancer (Table 2). The scatter plots for the causal relationship between gut microbiota and gastric cancer was presented in Figure 4. Specifically, the IVW analysis showed a protective effect against gastric cancer for *Clostridium sensustricto1* (OR = 0.540, 95%CI: 0.354–0.823, *p* = 0.004), *Actinomycetales* (OR = 0.756, 95%CI: 0.613–0.932, *p* = 0.009), *Rikenellaceae* (OR = 0.863, 95%CI: 0.746–0.999, *p* = 0.048), *Selenomonadales* (OR = 0.816, 95%CI: 0.666–1.000, *p* < 0.05), and *Negativicutes* (OR = 0.816, 95%CI: 0.666–1.000, *p* < 0.05). Conversely, *Roseburia* (OR = 1.342, 95%CI: 1.071–1.681, *p* = 0.011), *Family XI* (OR = 1.132, 95%CI: 1.012–1.267, *p* = 0.030), and *Eubacterium brachy group* (OR = 1.207, 95%CI: 1.074–1.355, *p* = 0.002) were linked to an increased risk of gastric cancer (Table 2; Figure 5).

### 3.3 Sensitivity analyses

Cochran’s Q statistic and Rucker’s Q statistic analyses indicated no heterogeneity in MR analyses for *Roseburia*, *Actinomycetales*, *Family XI*, *Rikenellaceae*, *Selenomonadales*, *Eubacterium brachy group*, *Negativicutes*, and *Actinomycetaceae* with gastric cancer (*p* > 0.05). However, *Clostridium sensustricto1’s* MR analysis with gastric cancer showed heterogeneity (*p* < 0.05). The MR Egger intercept test suggested no horizontal pleiotropy in the MR analyses across all examined taxa (*p* > 0.05) (Table 3). The “Leave one out” analysis further confirmed the robustness of our MR findings, showing that no single SNP disproportionately influenced the causal inference. Moreover, the MR-PRESSO global test corroborated the absence of horizontal pleiotropy across all taxa (*p* > 0.05), and the distortion test affirmed no outliers were present in our MR analyses (Table 3).

**Table 3:** Sensitivity analysis of the MR analysis results of exposures and outcomes.

Exposure	Outcome	Heterogeneity test		Pleiotropy test	MR-PRESSO	
		Cochran’s Q Test (*p* value)	Rucker’s Q Test (*p* value)	Egger Intercept (*p* value)	Distortion test	Global test
		IVW	MR-Egger	MR-Egger	Outliers	*p* value
Genus Clostridium sensustricto1	Gastric cancer	<0.001	0.020	0.161	NA	0.06
Genus Roseburia	Gastric cancer	0.135	0.113	0.547	NA	0.19
Order Actinomycetales	Gastric cancer	0.379	0.267	0.693	NA	0.43
Family Family XI	Gastric cancer	0.264	0.294	0.303	NA	0.4
Family Rikenellaceae	Gastric cancer	0.554	0.602	0.218	NA	0.61
Order Selenomonadales	Gastric cancer	0.570	0.488	0.764	NA	0.58
Genus *Eubacterium brachy* group	Gastric cancer	0.487	0.683	0.133	NA	0.62
Class Negativicutes	Gastric cancer	0.570	0.488	0.764	NA	0.64
Family Actinomycetaceae	Gastric cancer	0.378	0.266	0.691	NA	0.38

## 4 Discussion

In our investigation employing the Mendelian Randomization (MR) methodology, we explored the genetic causal connections between nine gut microbiotas and gastric cancer. This approach allowed us to conduct a causal analysis from a genetic standpoint, circumventing the limitations often encountered in traditional observational studies. Our findings identified *Clostridium sensustricto1*, *Actinomycetales*, *Rikenellaceae*, *Selenomonadales*, *Negativicutes*, and *Actinomycetaceae* as having a protective role against GC, while *Roseburia*, *Family XI*, and *Eubacterium brachy group* were associated with an increased risk at the genetic level. The MR analysis thus unveils the genetic causal relationships between these gut microbiotas and GC, highlighting the significance of the gut microbiome’s composition in influencing GC risk.

*Clostridium sensustricto1* are primarily strictly anaerobic, fermentative bacteria, one of the important anaerobic bacteria in the human gut (Spring et al., 2003). They metabolize various compounds such as carbohydrates, amino acids, alcohols and purines. Butyric acid is a “genus-specific” product of fermentation. 5 Various concentrations of acetic acid, lactic acid and/or ethanol, propanol or butanol can also be formed as fermentation products (Alou et al., 2018). Previous studies have documented *Clostridium’s* dual role in the digestive tract, capable of breaking down fat into secondary bile acids for carcinogenesis and fiber into butyrate for antitumor effects. The hypothesis that *Clostridium sensustricto1* mitigates GC pathogenesis through their complex metabolites warrants further investigation. The *Actinobacteria* order and the *Actinobacteriaceae* family, both *filamentous Gram-positive bacteria*, are recognized for their protective role against GC. *Actinomyces*, a well-known probiotic, has been shown to prevent constipation, improve intestinal function, aid in nutrient digestion and absorption, and produce vital nutrients (Ding et al., 2020). Wang et al. (2022) observed a significant reduction in actinomycetes abundance in patients with gastritis infected with *Helicobacter pylori* compared to uninfected individuals. Given *H. pylori’s* established role as a major GC risk factor, its infection may disrupt the original flora balance and diminish the protective effect of normal flora like *Actinomycetes*, which indirectly confirms our findings (Devi et al., 2021; Iino and Shimoyama, 2021). Furthermore, *Rikenellaceae* and other bacteria like *Selenomonadales* and *Negativicutes*, which belong to the phylum of *Firmicutes*, contribute significantly to the human intestinal flora and produce short-chain fatty acids (SCFA) (Mirzaei et al., 2021). Hu et al. (2018) observed a reduction in the pathways responsible for short-chain fatty acids (SCFAs) production in gastric cancer, indicating a heightened presence of inflammation and microbial imbalance within such pathological states. Furthermore, the gut microbiome and its metabolic by-products are known to influence the immune response to gastric cancer. The interaction between the microbiota and the immune system is mediated by pattern recognition receptors (PRRs) on innate immune cells. These receptors identify and differentiate between beneficial and detrimental bacteria through the detection of pathogen-associated molecular patterns (PAMPs), including bacterial endotoxins or lipopolysaccharides (Nasr et al., 2020). Various cells within the gut lumen can transport gut microbiota to engage with specific PRRs, triggering T or B cell-mediated responses (Wang et al., 2023). It has also been documented that *Helicobacter pylori* (Hp) can disrupt CD4 + T cell proliferation and diminish the production of IL-2 and IFN-g by enhancing programmed cell death-ligand 1 (PD-L1) expression on gastric epithelial cells (Das et al., 2006). Additionally, the presence of *Methylobacterium* in gastric cancer tissues has been linked to the suppression of CD8+ tissue-resident memory T cells (TRM), alongside a reduction in TGF-b expression (Peng et al., 2022). The gut microbiota, therefore, not only modulates immune responses during the development of tumors but also its metabolites significantly influence cancer progression and the immune system (Wang et al., 2023). Legoux et al. (2019) discovered that the metabolite 5-(2-oxopropylideneamino)-6-d-ribitylaminouracil fosters the proliferation of mucosal-associated invariant T (MAIT) cells from mucosal sites to the thymus, playing a crucial role in bolstering the body’s protective immune response. This study posits that *Roseburia*, *Family XI*, and *Eubacterium brachy group* contribute to the risk of gastric carcinogenesis. However, literature lacks comprehensive reports on this matter, highlighting the necessity for detailed investigations to clarify their roles.

This study’s strengths include being the first MR analysis to investigate the potential causal connection between gut microbiota and GC, utilizing the largest GWAS summary data on gut microbiota to date. Despite its novel insights, limitations exist, such as the use of summary statistics rather than raw data, limiting further subgroup analyses and the generalizability of findings across different populations and taxonomic levels. The majority of participants in the GWAS were of European descent. The use of 16S rRNA gene sequencing in the MiBioGen consortium’s GWAS data on gut microbiota only allows for the detection of genetic data at the genus to phylum level, and there is no genetic data for the species level. In addition, the selection of SNPs based on a predefined significance threshold may not capture the full genetic influence on GC risk, highlighting the need for caution in interpreting the results and the potential for unknown confounding factors.

## 5 Conclusion

Our studies have shown that there is a causal relationship between intestinal flora and gastric cancer at the genetic level. *Clostridium sensustricto1*, *Actinomycetales*, *Rikenellaceae*, *Selenomonadales*, *Negativicutes*, and *Actinomycetaceae* as having a protective role against GC, while *Roseburia*, *Family XI*, and *Eubacterium brachy group* were associated with an increased risk.

## Data availability statement

The original contributions presented in the study are included in the article/supplementary material, further inquiries can be directed to the corresponding author/s.

## Ethics statement

Ethical approval was not required for the study involving humans in accordance with the local legislation and institutional requirements. Written informed consent to participate in this study was not required from the participants or the participants’ legal guardians/next of kin in accordance with the national legislation and the institutional requirements.

## Author contributions

YC: Conceptualization, Data curation, Formal analysis, Methodology, Supervision, Writing – review & editing, Funding acquisition, Validation. GG: Conceptualization, Investigation, Project administration, Resources, Software, Supervision, Validation, Writing – original draft. CF: Conceptualization, Data curation, Formal analysis, Methodology, Software, Supervision, Writing – original draft, Writing – review & editing.

## Footnotes

^1^ www.mibiogen.org
